# Supplementary material for: Association between oral microbiome and seven types of cancers in East Asian population: a two-sample Mendelian randomization analysis
Source: Front Mol Biosci. 2023 Nov 21;10:1327893. doi: 10.3389/fmolb.2023.1327893 (PMC10702768; doi:10.3389/fmolb.2023.1327893)
Supplement: Supplementary file 1 [file DataSheet1.ZIP › Supplementary Materials/MR plots of saliva/saliva═╝/Lung Cancer/pheno.2346_to_lung cancer_forest.pdf]

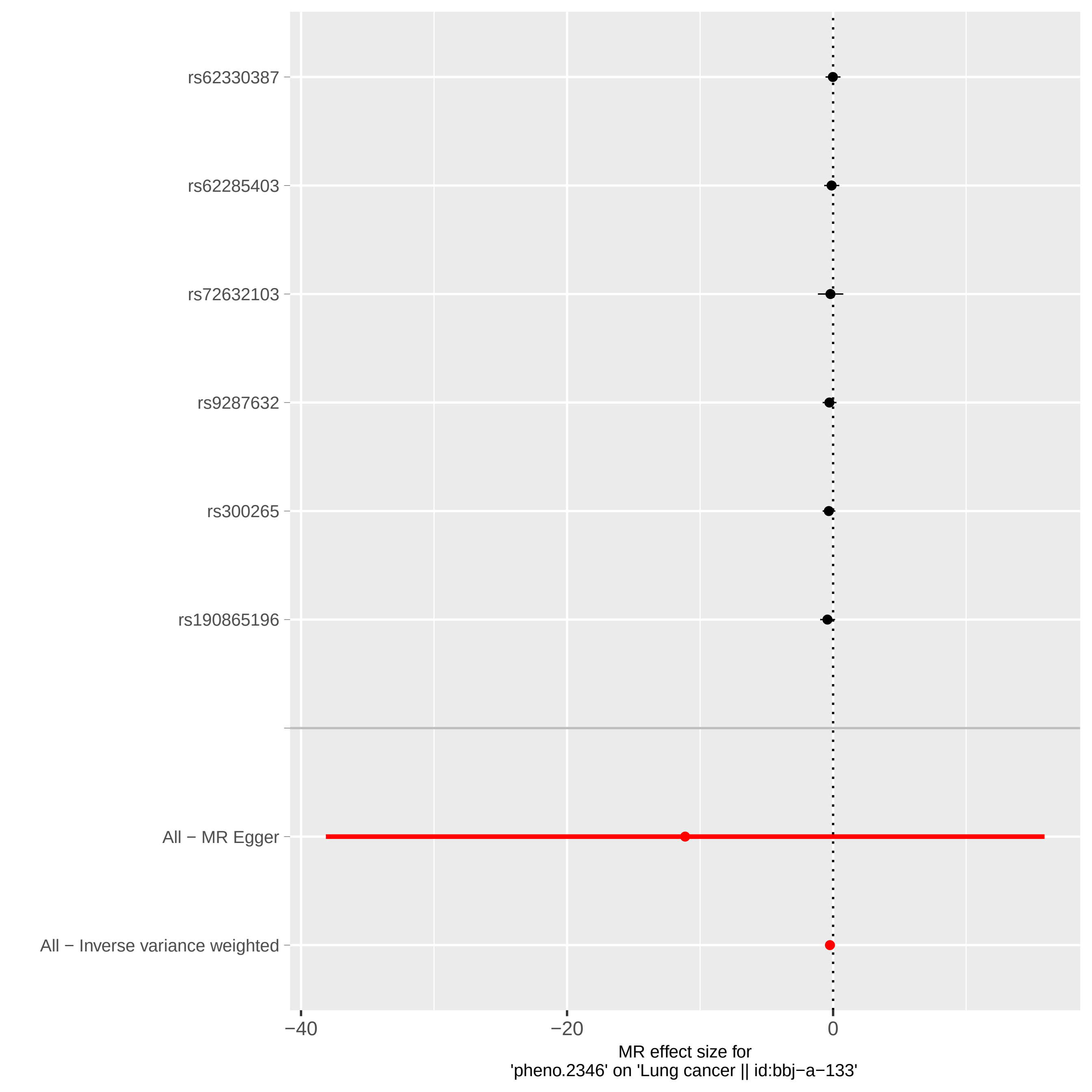
rs62330387
rs62285403
rs72632103
rs9287632
rs300265
rs190865196
All − MR Egger
All − Inverse variance weighted
−40
−20
0
MR effect size for
'pheno.2346' on 'Lung cancer || id:bbj−a−133'
